# Use of atrial septal occluder in the treatment of chronic fistula following post-esophagectomy anastomotic leak

**DOI:** 10.1055/a-2134-8709

**Published:** 2023-08-23

**Authors:** Manisha Daminda Kariyawasam, Jonathan Liang Yap, Zehao Tan, Tiffany Lye, Weng Hoong Chan, Jeremy Tian Hui Tan, Chin Hong Lim

**Affiliations:** 1 Department of Upper Gastrointestinal and Bariatric Surgery, Singapore General Hospital, Singapore; 2 Department of Cardiology, National Heart Center Singapore, Singapore; 3 Department of Vascular & Interventional Radiology, Singapore General Hospital, Singapore

Therapeutic endoscopy plays a key role in the management of gastrointestinal fistulae. Endoscopic interventions with stents 1 , endoscopic clips, vacuum therapy 2 , etc. have revolutionized the management of post-surgical anastomotic leaks.

A 66-year-old man diagnosed with gastro-esophageal junction adenocarcinoma underwent a minimally invasive Ivor Lewis esophagectomy with gastric pull-up. The postoperative phase was uneventful. The patient presented 8 weeks after surgery with a respiratory tract infection while receiving adjuvant chemotherapy. A computed tomography (CT) scan revealed a right paramediastinal abscess due to an anastomotic leak. Over a period of 6 months, the patient underwent CT-guided percutaneous drain placement, video-assisted thoracoscopic debridement of empyema, endoscopic insertion of a fully covered metal stent (Niti-S MEGA Esophageal Stent; TaeWoong Medical, Gyeonggi-do, South Korea), and internal drainage with double-pigtail stents. Despite these measures, the defect failed to close, resulting in recurrent chest infections and a persistently discharging sinus at the back of the right chest. We decided to proceed with fistula closure with an atrial septal occluder device 3 4 .

Following general anesthesia, the patient was positioned in the left lateral position. The first endoscopist passed a 5-mm gastroscope (GIF-XP160; Olympus, Tokyo, Japan) through the external opening of the fistula to reach the intrathoracic abscess cavity, and the defect was identified with a double-pigtail stent in situ ( Video 1 ). The second endoscopist then introduced the 10-mm gastroscope (GIF-XP160; Olympus) transorally and a guidewire was introduced through the defect. Under direct visualization, the Amplatzer Cribriform Multi-Fenestrated Septal Occluder (AGA Medical Corporation, Plymouth, Minnesota, USA) with its delivery system was introduced along the guidewire into the abscess cavity 5 . The left atrial (LA) disc followed by the right atrial (RA) disc were deployed under endoscopic ( Fig. 1 ) and fluoroscopic guidance ( Fig. 2 ). The procedure time was 90 minutes.

**Video 1** Endoscopic closure of chronic gastrointestinal fistula using atrial septal occluder device.


**Fig. 1 a:**
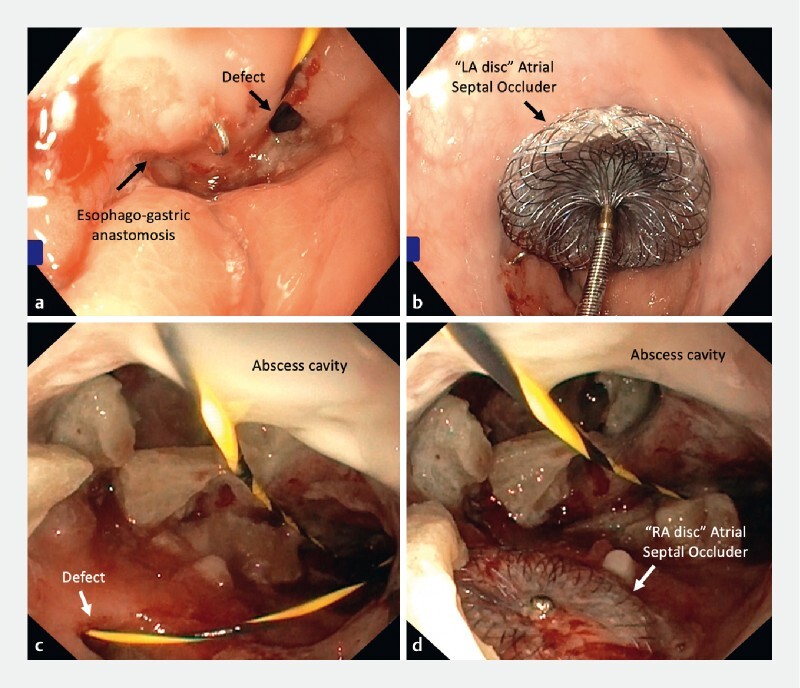
Defect at the esophagogastric anastomosis with guidewire in situ. **b** Left atrial disc of the atrial septal occluder fully deployed. **c** Anastomotic defect visualized inside the abscess cavity. **d** Right atrial disc of atrial septal defect deployed inside the abscess cavity.

**Fig. 2:**
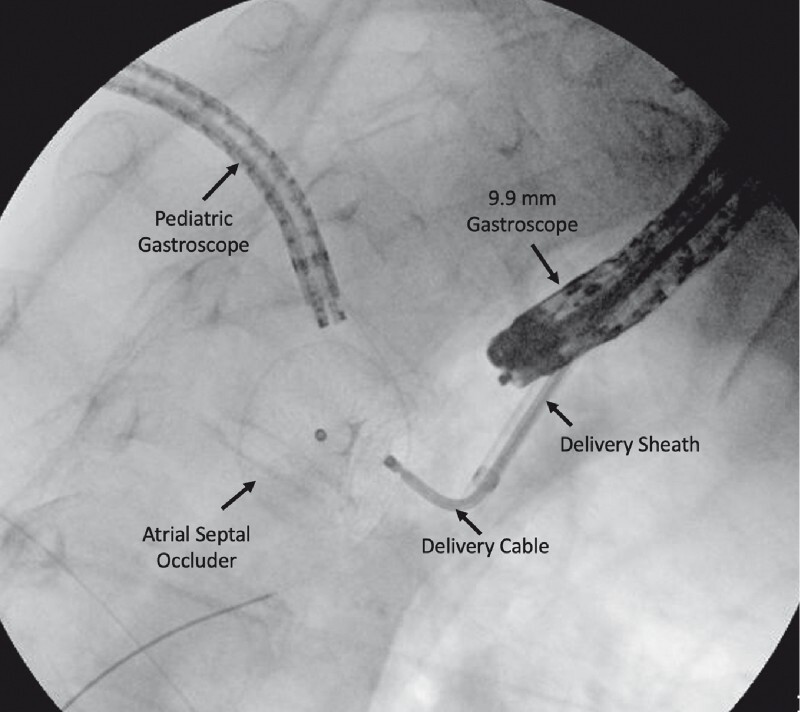
Fluoroscopic view of deployment of the atrial septal occluder.

CT imaging with oral contrast at 1 week and 8 weeks post-procedure showed no extravasation of contrast through the anastomotic defect ( Fig. 3 ). The nasojejunal feeding tube was removed and at 3 months the patient is tolerating a liquid to pureed diet.

**Fig. 3:**
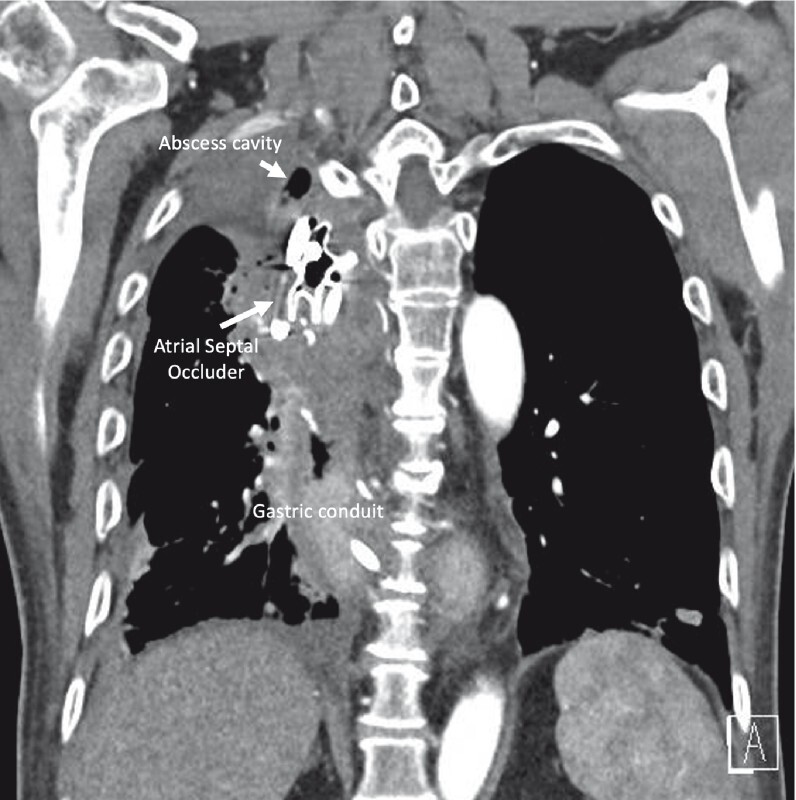
Interval computed tomography scan at 1 week showing the atrial septal occluder device positioned between the abscess cavity and neo-esophagus.

Endoscopy_UCTN_Code_TTT_1AO_2AI

## Notes

Endoscopy E-Videos

https://eref.thieme.de/e-videos
*E-Videos* is an open access online section of the journal *Endoscopy* , reporting on interesting cases and new techniques in gastroenterological endoscopy. All papers include a high-quality video and are published with a Creative Commons CC-BY license. Endoscopy E-Videos qualify for HINARI discounts and waivers and eligibility is automatically checked during the submission process. We grant 100% waivers to articles whose corresponding authors are based in Group A countries and 50% waivers to those who are based in Group B countries as classified by Research4Life (see: https://www.research4life.org/access/eligibility/ ). This section has its own submission website at https://mc.manuscriptcentral.com/e-videos
